# Characterization of sugarcane (*Saccharum* spp.) leaf senescence: implications for biofuel production

**DOI:** 10.1186/s13068-016-0568-0

**Published:** 2016-07-22

**Authors:** Maria Thereza Bazzo Martins, Wagner Rodrigo de Souza, Bárbara Andrade Dias Brito da Cunha, Marcos Fernando Basso, Nelson Geraldo de Oliveira, Felipe Vinecky, Polyana Kelly Martins, Patrícia Abrão de Oliveira, Bruna Cersózimo Arenque-Musa, Amanda Pereira de Souza, Marcos Silveira Buckeridge, Adilson Kenji Kobayashi, Betania Ferraz Quirino, Hugo Bruno Correa Molinari

**Affiliations:** Genetics and Biotechnology Laboratory, Embrapa Agroenergy (CNPAE), Brasília, DF 70770-901 Brazil; Genomic Sciences and Biotechnology Program, Universidade Catolica de Brasilia, Brasília, DF 70790‑160 Brazil; Biomass and Biofuel Chemistry Laboratory, Embrapa Agroenergy (CNPAE), Brasília, DF 70770-901 Brazil; Laboratory of Plant Physiological Ecology (LAFIECO), Department of Botany-Institute of Biosciences, University of São Paulo, São Paulo, SP 05508-090 Brazil

**Keywords:** Sugarcane, Nutrient remobilization, Natural leaf senescence, Cell wall, Lignocellulosic ethanol, Renewable energy, Biotechnology

## Abstract

**Background:**

Second-generation ethanol (2G-bioethanol) uses lignocellulosic feedstocks for ethanol production. Sugarcane is one among the most suitable crops for biofuel production. Its juice is extracted for sugar production, while sugarcane bagasse, straw, and senescing leaves are considered industrial waste. Senescence is the age-dependent deterioration of plant cells, ultimately leading to cell death and completion of the plant life cycle. Because senescing leaves may also be used for biofuel production, understanding the process of natural senescence, including remobilization of nutrients and its effect on cell walls can provide useful information for 2G-bioethanol production from sugarcane leaves.

**Results:**

The natural senescence process in leaves of the commercial sugarcane cultivar RB867515 was investigated. Senescence was characterized by strong reduction in photosynthetic pigments content, remobilization of the nutrients N, P, K, B, Cu, Fe, and Zn, and accumulation of Ca, S, Mg, B, Mn, and Al. No significant changes in the cell-wall composition occurred, and only small changes in the expression of cell wall-related genes were observed, suggesting that cell walls are preserved during senescence. Senescence-marker genes, such as *SAG12*-like and *XET*-like genes, were also identified in sugarcane and found to be highly expressed.

**Conclusions:**

Our study on nutrient remobilization under senescence in a vigorous sugarcane cultivar can contribute to the understanding on how nutrient balance in a high-yielding crop is achieved. In general, neutral monosaccharide profile did not change significantly with leaf senescence, suggesting that senescing leaves of sugarcane can be as a feedstock for biofuel production using pretreatments established for non-senescing leaves without additional efforts. Based on our findings, the potential biotechnological applications for the improvement of sugarcane cultivars are discussed.

**Electronic supplementary material:**

The online version of this article (doi:10.1186/s13068-016-0568-0) contains supplementary material, which is available to authorized users.

## Background

The use of ethanol from renewable sources is extremely important to reduce greenhouse gas emissions and dependence on fossil fuels [1]. Biofuels are less polluting with respect to emission of sulfur, lead, and greenhouse gases (CO_2_ and CH_4_) [2]. Thus, there is a tendency to increase the demand for ethanol by increasing the use of biofuels. Several efforts have been made to develop alternative technologies for ethanol production from lignocellulosic biomass [3–5]. This technology, called second-generation ethanol (2G-bioethanol) production, aims to disrupt plant cell-wall polysaccharides, such as cellulose and hemicelluloses into smaller fermentable sugars and utilize them to produce ethanol [6, 7]. However, lignocellulosic biomass is highly recalcitrant to deconstruction due to the rigid and compact structure of the plant cell wall [5, 8, 9]. Nowadays, the disruption of cell walls during 2G-bioethanol production is made mainly using chemical or enzymatic pretreatments, which increases significantly the cost of this process [7, 10, 11].

Brazil is the largest sugarcane producer worldwide, and its production is intended mainly for ethanol and sugar production [12]. According to CONAB [13] and UNICA [14], Brazil is also the largest producer of ethanol from biomass, through sucrose fermentation. Furthermore, the potential for 2G-bioethanol production using the residue materials, such as bagasse and leaves, is enormous for sugarcane. Currently, the biomass left in the form of bagasse and leaves has the potential to increase ethanol production by approximately 40 % [7]. However, one of the key problems to release the sugars from cell walls for fermentation has been recalcitrance, i.e., the difficulty to access all the linkages among carbohydrates and phenylpropanoids that need to be broken to hydrolyze the cell walls [7, 9, 15, 16].

Recently, Tavares et al. [17] and Grandis et al. [18] proposed that endogenous degradation systems could be used to help decrease cell-wall recalcitrance and facilitate production of 2G-bioethanol. Some natural cell wall degrading processes may occur in plant cells, and such degradation appears to be preceded by programmed cell death (PCD). Because senescence is a type of PCD, the understanding of senescence-related processes could lead to the discovery of genes related to such mechanisms, and these could be used to engineer plants that would induce tissues to modify their own walls thus facilitating 2G ethanol production.

Senescence is the age-dependent deterioration of plant cells, ultimately leading to cell death and completion of the plant life cycle. In addition to the natural senescence process, biotic and abiotic factors are known to activate senescence pathways [19]. Leaf senescence constitutes the final stage of leaf development, and the earliest and most important event during this process is the chloroplast degradation [20, 21]. Consequently, a decrease in the photosynthetic rate and chlorophyll content occurs, with subsequent leaf yellowing [22]. During senescence, some compounds released from degraded chloroplasts and other organelles are remobilized to other developing tissues, such as young leaves or fruits, and grains [20, 21]. Senescence is a tightly controlled process, and, therefore, many senescence-associated genes are up-regulated during this process [19, 23]. To our knowledge, the senescence process in sugarcane has not been investigated so far. We believe that the study of the sugarcane leaf senescence can contribute not only to the understanding of this important natural process, but also to the development of new strategies to control it, improving agricultural traits of this crop. For example, studying the dynamics of synthesis and degradation of cell wall during sugarcane senescence might help in the development of biotechnological tools to assist in the bioethanol production from lignocellulosic biomass [8, 17, 24]. Moreover, plant senescence pathways can be activated by biotic and abiotic stresses, and its understanding under natural conditions offers a suitable strategy to generate transgenic crops able to cope with these stresses.

The aim of the current work was the analysis of photosynthetic pigments, nutrient remobilization, and cell-wall modifications during the natural leaf senescence process in the sugarcane cv. RB867515. This cultivar has widely been used in all producing regions in Brazil, and it is characterized by drought stress tolerance, high sugar content and yield, fast growth, and great potential for lignocellulosic ethanol production [25]. We have measured these parameters not only in different leaves (from non-senescing to senescing ones), but also at different regions of the same leaf (base, middle, and tip of the leaf blade). Leaf senescence was characterized in sugarcane by reduction in photosynthetic pigment content, remobilization of some nutrients, and accumulation of others, but no significant changes in the cell-wall composition, paralleled by small changes in the cell wall-related gene expression.

## Methods

### Plant material and sampling

**Fig. 1:**
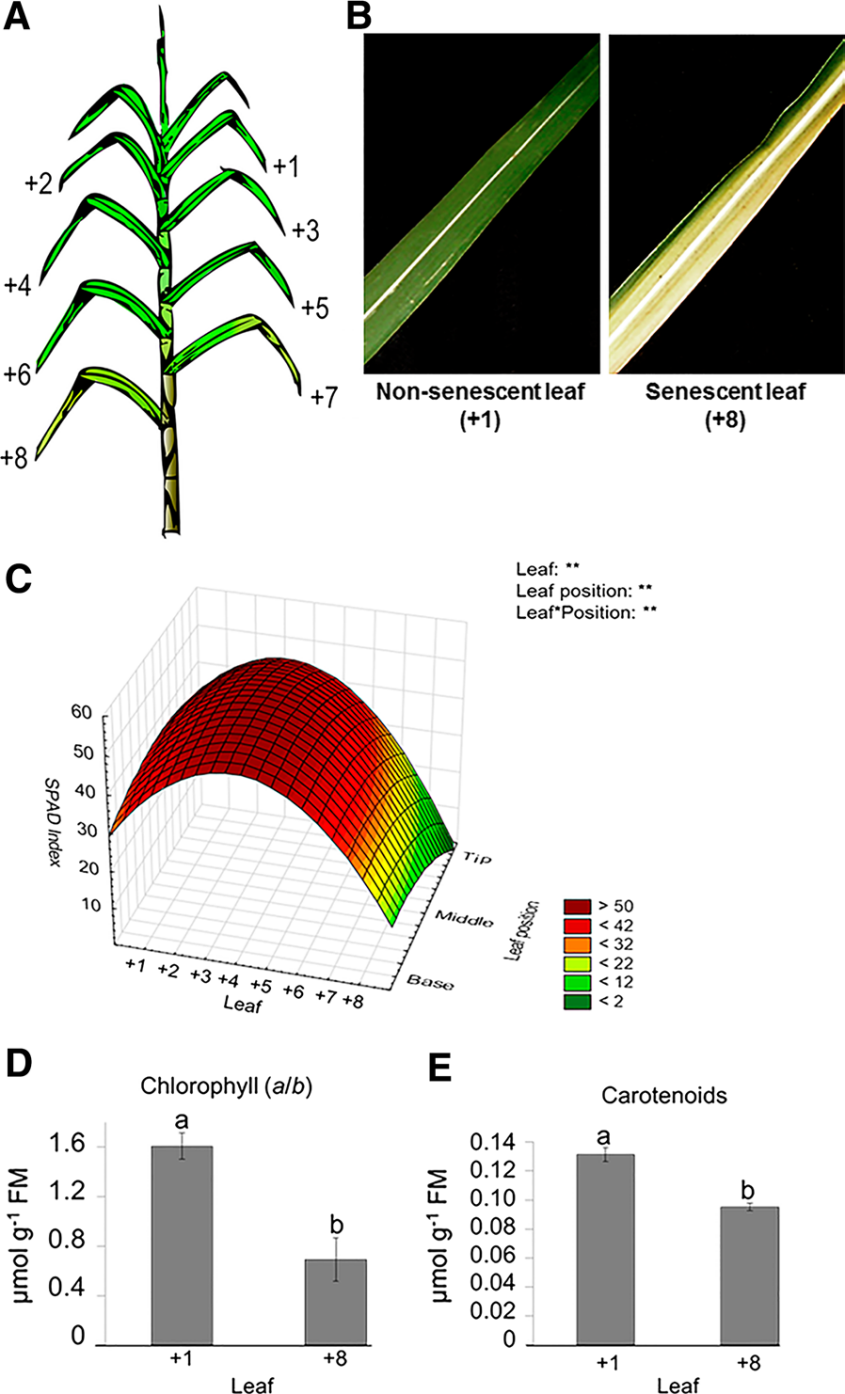
Photosynthetic pigment content in sugarcane leaves cv. RB867515. **A** Representative scheme of a sugarcane plant depicting the leaf senescence gradient. Leaves were numbered according to the system proposed by Kuijper [26]. **B** Non-senescent (+1) and senescent (+8) leaves of 8-month-old sugarcane cv. RB867515. **C** Three-dimensional plot of the SPAD index changes in the ‘between-leaves’ gradient and the ‘in-leaf’ gradient (*n* = 5; read *per* position/leaf = 3); Statistical analysis: leaf refers to statistics applied to different leaves (from +1 to +8); leaf position refers to statistics applied to the same leaf (base, middle and tip portions) and leaf *position refers to statistics applied to the whole data set (‘between-leaves’ and ‘in-leaf’ gradients); asterisk indicates statistical difference: **p ≤ 0.01, *p ≤ 0.05, and *ns* non-significant; The ANOVA and significant *p* values are available in the Additional file 2. **D** Chlorophyll *a*/*b* ratio and **E** carotenoids content in sugarcane leaves; Statistical differences (*p* ≤ 0.05) were obtained with ANOVA followed by Tukey’s test; *Different letters* indicate statistical significance at *p* ≤ 0.05. *FM* fresh mass. *Vertical bars* show ± S.E. for *n* = 3

Plants of the sugarcane cv. RB867515 (*Saccharum* spp.) provided by Inter-University Network for Development of the Sugarcane-Ethanol Sector (RIDESA, Brazil) were maintained under field conditions at Embrapa Cerrados, from April, 2008 to December, 2008 (Planaltina, DF, Brazil; Latitude 15°36′10.7″ and Longitude 47°42′37.7″). The climate is classified as Aw type (tropical savannah; Köppen-Geiger) and is characterized by a long drought period. The soil of the experimental area was chemically analyzed and corrected with lime (2 Mg ha^−1^ of dolomitic limestone), gypsum (3 Mg ha^−1^), and fertilization with nitrogen (N) 20 kg/ha, phosphorus (P_2_O_5_) 150 kg/ha, and potassium (K_2_O) 80 kg/ha using the chemical fertilizer NPK 04-30-16. Seven months after planting, top dressing was carried out with N 100 kg/ha, P_2_O_5_ 50 kg/ha, and K_2_O 100 kg/ha, using chemical fertilizer NPK 20-5-20. A leaf senescence gradient was harvested from 8-month-old plants and evaluated using the leaf numbering system proposed by Kuijper [26] (Fig. 1A). The first completely expanded leaf with visible auricle and photosynthetically active was considered as +1 leaf. In addition, to evaluate the in-leaf senescence gradient, leaves were divided in three parts: base, middle, and tip positions along the leaf blade. All analyses were conducted using plant cane.

### Photosynthetic pigments content

Total chlorophyll content in +1 to +8 leaves was measured with a portable optical chlorophyll meter (SPAD-502; Minolta Corporation, Tokyo, Japan) using five replicates *per* leaf and three readings *per* position of the leaf blade (+1 to +8 leaves at base, middle, and tip positions), and it was represented as SPAD index [27]. In addition, for +1 and +8 leaves, Chl-*a*/*b* ratio and carotenoids (Cars) contents were also determined after acetone extraction as described by Henry and Grime [28]. The Chl-*a*/*b* ratio and Cars content estimation were performed using extinction coefficients and equations proposed by Lichtenthaler [29].

### Leaf nutrient concentration

To estimate the content of nutrients in sugarcane plants, we used the base portions of the +3 leaf blade, which is the leaf commonly used to evaluate this parameter in sugarcane [30]. The macro and micronutrients contents present in the +3 leaf were obtained from three biological replicates each composed of a leaf pool of 5 plants.

Macro and micronutrients concentrations along the leaf gradient, phosphorous (P), potassium (K), calcium (Ca), magnesium (Mg), sulfur (S), boron (B), copper (Cu), iron (Fe), manganese (Mn), zinc (Zn), and aluminum (Al) were obtained from leaf tissue (+1 to +8 leaves, each leaf blade was divided into base, middle, and tip portions) of each replicate (three replicates, each replicate consisting of five bulks collected from five different plants). The nutrient concentration profile was obtained from 1 g of dry mass processed by acid digestion method as described by Adler and Wilcox [31] and determined by optical emission spectrometry with inductively coupled argon plasma in Thermo Jarrell Ash spectrometer model IRIS/AP, as described by Murad et al. [25]. Leaf nitrogen concentration was measured by colorimetry using the distillation method in Kjeldahl semi-micro apparatus, as described by Persson et al. [32].

### Neutral monosaccharide composition

Leaves +1 to +8 were divided into base, middle, and tip portions of the leaf blade, and each replicate consisted of five different plants. All analyses were based on the procedures described by De Souza et al. [33]. The material was freeze-dried and ground into a fine powder in a ball mill. Five hundred milligrams of each sample were subjected to six consecutive extractions with 25 mL of 80 % (v/v) ethanol at 80 °C for 20 min. Each extraction was followed by centrifugation (10 min at 8500*g*), and the supernatant was discarded to remove the soluble sugars. The absence of soluble sugars was confirmed by the phenol–sulfuric method described by Dubois et al. [34]. The cell-wall extract samples were hydrolyzed with 2 M trifluoroacetic acid (TFA) for 1 h at 120 °C. The acid was evaporated under vacuum and the monosaccharides were resuspended in 2 mL of ultra-purified water. Neutral monosaccharide profile was analyzed by high-performance anion exchange chromatography with pulsed amperometric detection (HPAEC-PAD) on a CarboPac SA10 column (DX-500 system, Dionex™) using a mixture of 99.2 % water and 0.8 % (v/v) 150 mM NaOH as eluent (1 mL min^−1^). The monosaccharides were detected with a post-column addition of base with 500 mM NaOH (1 mL min^−1^). The relative proportion of each neutral monosaccharides was calculated based on the peak area, considering the sum of the peak areas of all monosaccharides as 100 %.

### Statistical analysis

The data of the SPAD index (Chl-*a*/*b* ratio), macro and micronutrients, and neutral monosaccharides profile were analyzed for normality (Shapiro–Wilk test) and, accordingly, were compared using the *F* test, ANOVA and *Pearson*’s parametric correlation test, considering significant when *p* ≤ 0.05. The significant factors (nutrient concentration in the ‘between-leaves’ gradient and in the ‘in-leaf’ gradient) were represented in three-dimensional plots, according to leaf number and position of the blade leaf, using StatSoft version 10 [35]. The photosynthetic pigments content was analyzed with one-way ANOVA and Tukey’s test (*p* ≤ 0.05), using the JMP software, version 5.0.1 (SAS Institute Inc.). The apparent nutrient remobilization of macro and micronutrients was calculated by dividing the difference between the average of +1 and +8 leaves by the average of the +1 leaf (i.e., non-senescent leaf), and nutrient accumulation percentage was obtained by dividing the difference between the average of +8 and +1 leaves by the average of +8 leaf. To evaluate which factors (SPAD index, macro and micronutrients concentration, and neutral monosaccharide content) were likely to be more relevant to the natural process of sugarcane leaf senescence, a principal component analysis (PCA) was performed comparing all collected data. Each matrix was previously tested for normality by Anderson–Darling test (*p* < 0.05), for homogeneity of variance by Levene’s and Bartlett’s tests, and for symmetry given by *skewness* value allowing a variation between −1 and +1. A GLM test (General Linear Model) was performed to analyze if the level of the variation represented on the axes was related to senescence and the significance of each component.

### RNA isolation and real-time qPCR analysis

The +1 (non-senescing) and +8 (senescing) leaves were harvested at three time points during the day: 0800, 1300, and 1800 h. Harvested leaves were immediately frozen in liquid nitrogen and stored at −80 °C. RNA isolation was performed using TRIzol Reagent (Invitrogen Life Technologies), according to the manufacturer’s recommendations. The purity/concentration was measured using a NanoDrop ND-1000 spectrophotometer (A260/280 and A260/230 ratios). The high-quality and DNA-free total RNA was used as template for cDNA synthesis using the ThermoScript™ RT-PCR System Kit (Invitrogen, Carlsbad, CA), according to manufacturer’s instructions, and its concentration was diluted/adjusted in nuclease-free water and used for real-time PCR (RT-qPCR).

Four genes involved in cell-wall modification [α-arabinofuranosidase (*α*-*ARF*), α-xylosidase (*α*-*XYL*), β-glucosidase (*β*-*GLU*), and cellulase], and two involved in the leaf senescence [cysteine protease (*SAG12*-like) and xyloglucan endotransglucosylase (*XET*-like)] were selected to evaluate the expression profile of the targeted gene. The *SAG12*-like and *XET*-like genes were identified in the sugarcane genome as orthologues of the *SAG12* and *XET* of *Arabidopsis thaliana* [36, 37]. We decided to search for *SAG12* candidates in the sugarcane EST database (SUCEST; [38]), using the Arabidopsis *SAG12* gene as a query sequence. Subsequent BLAST analysis identified a region of high similarity between the Arabidopsis *SAG12* gene and the putative sugarcane *SAG12*-like gene; this sequence was used for primer design to determine the expression pattern of the *SAG12*-like gene sugarcane senescing leaves. The same strategy was applied to the genes for the cell wall modification; however, the sequence was based on previously characterized genes in *Sorghum bicolor,* a monocot phylogenetically similar to sugarcane.

The primers (Additional file 1: Table S1) were designed using the PrimerQuest tool (Integrated DNA Technologies, Coralville, IA, USA). Relative expression was determined on a 7500 Fast Real-Time PCR System (Applied Biosystems), using SYBR green (SYBR Green qPCR Mix-UDG/ROX, Invitrogen), following the manufacturer’s instructions. The relative quantification of the selected genes was determined by the 2^−ΔΔCt^ comparative method [39]. The data were analyzed with the SDS 2.1 software (Applied Biosystems) and normalized using the expression profile of the glyceraldehyde-6-phosphate dehydrogenase (GAPDH) endogenous gene [40, 41]. All gene expression profile data were analyzed by one-way ANOVA and the means were compared using Tukey’s test.

## Results

### Determination of the harvesting period and photosynthetic pigments content

Chloroplast degradation and subsequent loss of total chlorophyll are one of the earliest events during leaf senescence [28]. Therefore, the suitable harvesting period of sugarcane senescing and non-senescing leaves was determined by the analysis of total chlorophyll content throughout the plant life cycle using an SPAD meter. This method was chosen, because it is fast, accurate, and non-destructive. Measurements were taken in different leaves (between-leaves), named from +1 to +8 leaves, according to Kuijper numbering system (Fig. 1A), and in the different portions of the same leaf, named base, middle, and tip (in-leaf senescence gradient). During the eighth month, we observed a senescence gradient between +1 and +8 leaves, and also at different portions of the same leaf (Fig. 1B). Using the SPAD index to gauge senescence, we observed that the tip of the +8 leaf was the most senescent part of the tissue, while the base of the +1 leaf was the least senescent, indicating that in sugarcane, senescence proceeds from +8 to +1 leaves, and from the tip to the base of the leaf (Fig. 1C). Therefore, we decided to harvest leaf tissues for deeper analyses during the beginning of the eighth month of sugarcane growth.

For +1 and +8 leaves, the Chl-*a*/*b* ratio and carotenoids (Cars) content were measured using the acetone extraction method. As expected, senescing leaves (+8) presented the Chl-*a*/*b* ratio two-fold lower, compared with non-senescing (+1) leaves (Fig. 1D). These results are corroborated by the leaf yellowing observed in +8 leaves, while +1 leaves remained green at this stage (Fig. 1B). Similarly, Cars content decreased in senescing leaves (Fig. 1E), demonstrating a regular senescence pattern in sugarcane leaves.

### Nutrient remobilization and accumulation

The content of nutrients in sugarcane cv. RB867515 measured using the +3 leaf followed the order N > K >Ca > Mg = S > P for macronutrients, and Fe > Mn > Zn > Cu > B for micronutrients (Table 1).

**Table 1:** Estimated demand of macro and micronutrients in sugarcane index leaf cv. RB867515

Macronutrients (g kg^−1^ dry mass)		Micronutrients (mg kg^−1^ dry mass)	
Nitrogen	14.0 (±1.1)^a^	Iron	132.7 (±15.1)
Potassium	10.9 (±0.7)	Manganese	54.0 (±13.6)
Calcium	4.6 (±1.3)	Zinc	11.0 (±1.3)
Magnesium	2.4 (±0.1)	Copper	4.6 (±0.8)
Sulfur	2.2 (±0.3)	Boron	2.9 (±0.2)
Phosphorus	1.4 (±0.1)		

^a^Values of each nutrient are presented as mean ± standard deviation (three biological replicates, each composed of a +3 leaf pool of the 5 plants)

**Fig. 2:**
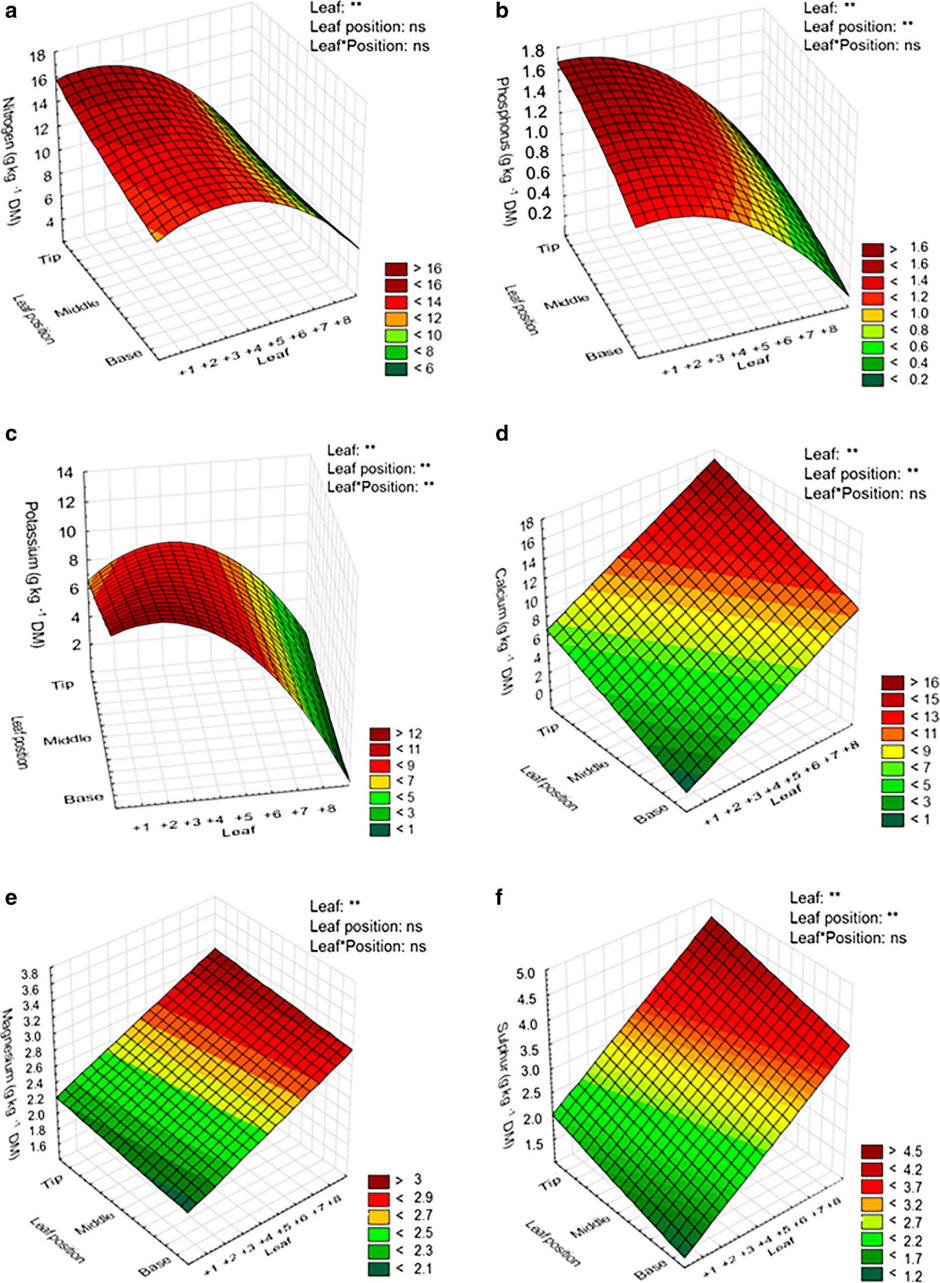
Three-dimensional plot of macronutrients content in the ‘between-leaves’ gradient and in the ‘in-leaf’ senescence gradient of the sugarcane cv. RB867515. Nitrogen (**a**), phosphorus (**b**), potassium (**c**), calcium (**d**), magnesium (**e**) and sulfur (**f**) were tested. The “leaf” axis represents the sugarcane leaves numbering system according to Kuijper [26]. *Asterisk* in the figure indicates statistical difference: **p ≤ 0.01, *p ≤ 0.05, and *ns* non-significant. The ANOVA and *p* values significant are available in the Additional file 2. *DM* dry mass

**Fig. 3:**
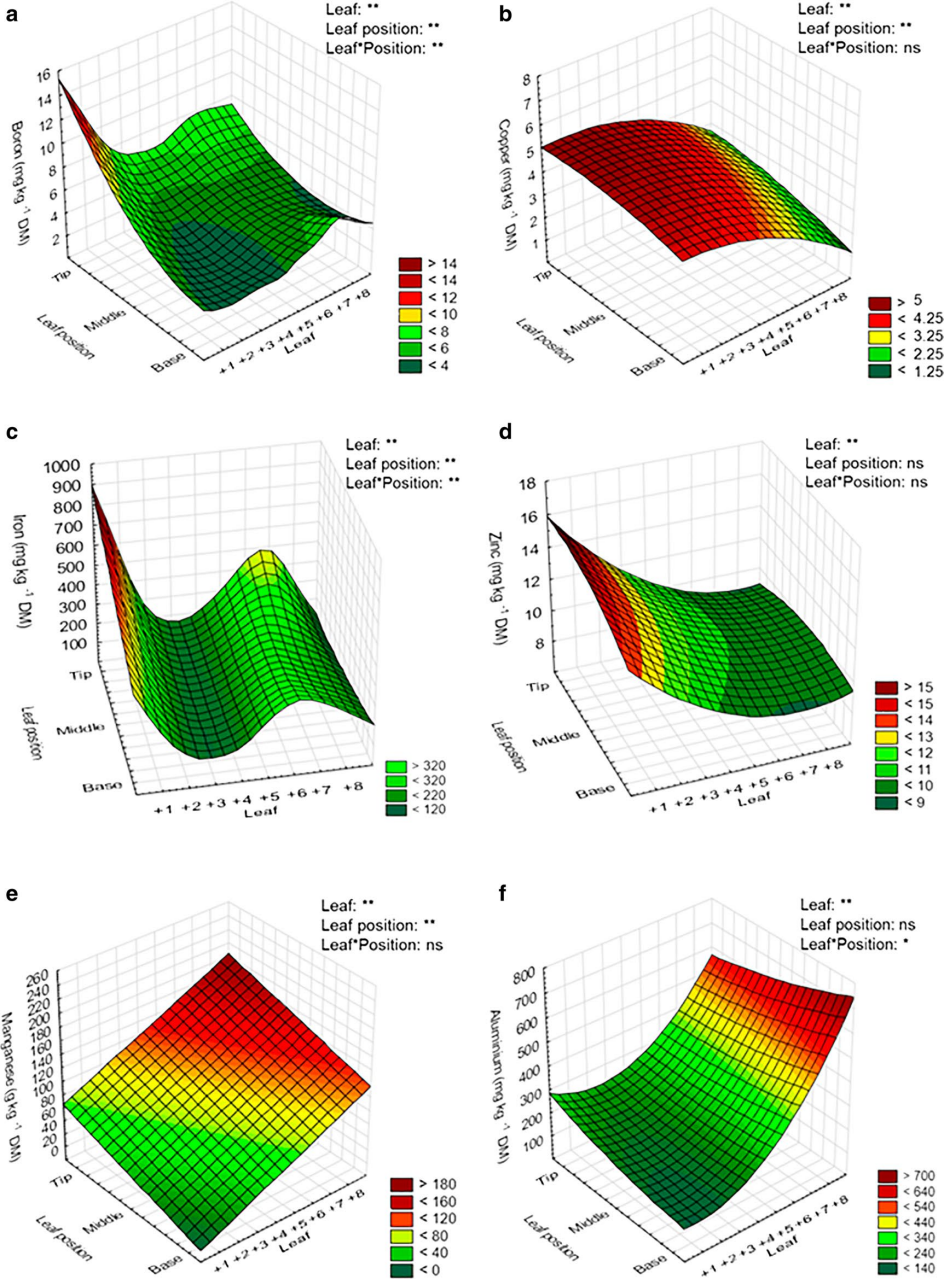
Three-dimensional plots of micronutrients content in the ‘between-leaves’ gradient and in the ‘in-leaf’ senescence gradient of the sugarcane cv. RB867515. Boron (**a**), copper (**b**), iron (**c**), zinc (**d**), manganese (**e**), and aluminum (**f**) were tested. The “leaf” axis represents the sugarcane leaves numbering system according to Kuijper [26]. Statistical analysis: leaf refers to statistics applied in different leaves (from +1 to +8); leaf position refers to statistics applied to the same leaf (base, middle and tip portions), and leaf *position refers to statistics applied to the whole data set (‘between-leaves’ and ‘in-leaf’ gradients); asterisk indicates statistical difference: **p ≤ 0.01, *p ≤ 0.05, and *ns* non-significant. The ANOVA and *p* values significant are available in the Additional file 2. *DM* dry mass

The levels of leaf macro (N, P, K, Ca, Mg and S) and micronutrients (Cu, Zn, Fe, Mn and B) were analyzed comparing leaves at different stages of senescence (‘between-leaves’ comparison) and different portions (‘in-leaf’ comparison) of sugarcane leaves. Three-dimensional plots showing the levels of macro (Fig. 2) and micronutrients (Fig. 3) showed significant differences between-leaves at different senescence stages (‘between-leaves’ gradient) which were observed for all nutrients. For the different parts of the leaf (‘in-leaf’ gradient), differences were observed for all nutrients with the exception of N, Mg, Zn, and Al. These results were corroborated by variance analysis of the macro and micronutrients, which demonstrated the significant differences in the ‘between-leaves’ (+1 to +8) and the ‘in-leaf’ (base, middle, tip portions) senescence gradients (Additional file 2). These data indicated either a high mobility or accumulation of these nutrients in sugarcane leaves.

**Fig. 4:**
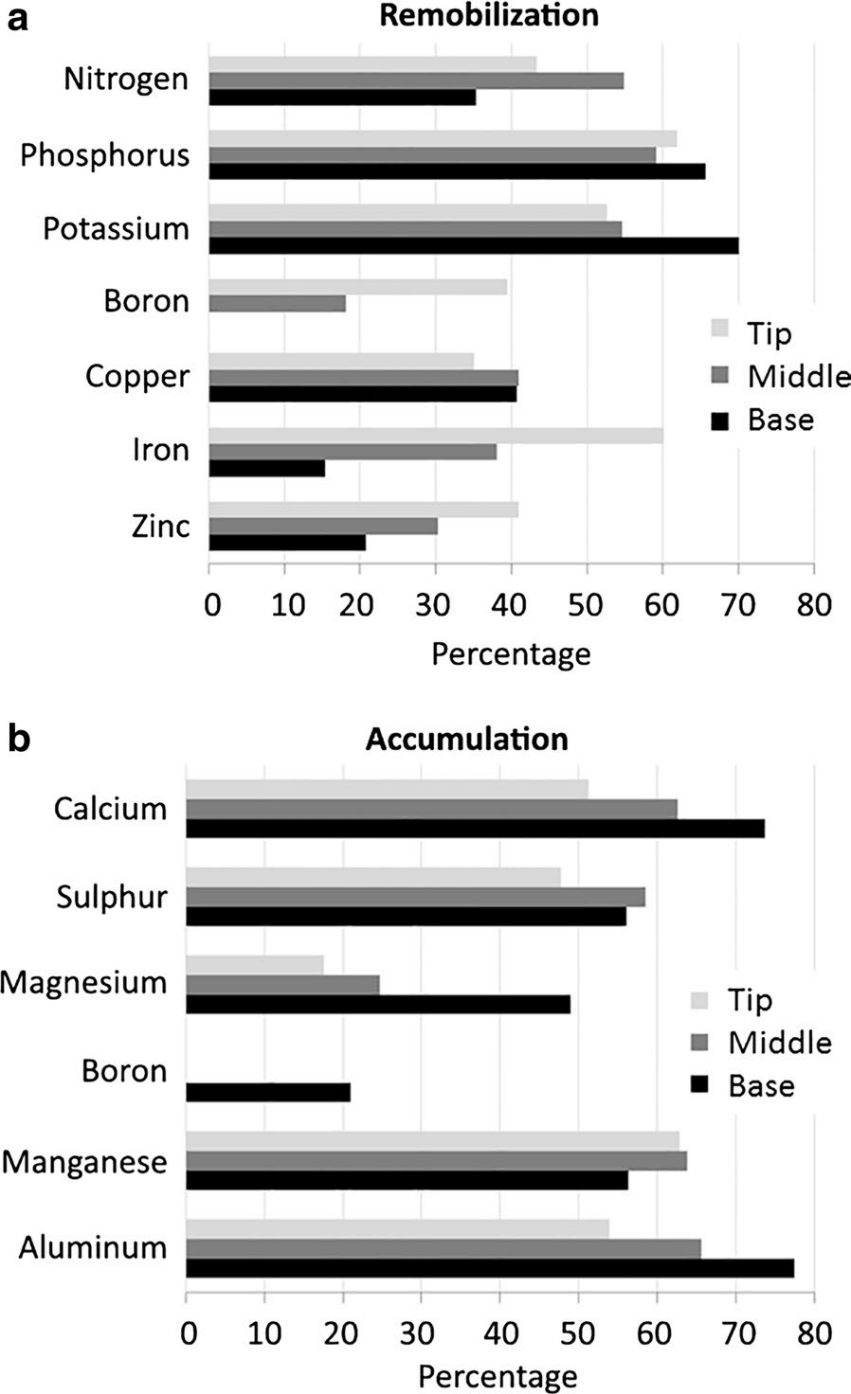
Apparent nutrient remobilization (**a**) and accumulation (**b**) of nutrients during senescence of leaves of the sugarcane cv. RB867515. The nutrient reduction percentage was calculated dividing the difference between the +1 and +8 leaves by +1 leaf. In contrast, the nutrients accumulation percentage was obtained by dividing the difference between the +1 and +8 leaves by +8 leaf

The nutrients remobilized during sugarcane leaf senescence are presented in Fig. 4a. N, P, and K were the nutrients that had the highest levels of remobilization, with values of 55, 65, and 70 % of remobilization, respectively. B, Cu, Fe, and Zn were also remobilized during leaf senescence, displaying levels of remobilization ranging from 20 to 40 %. In addition, the different portions of the leaf (base, middle, and tip) displayed different patterns of remobilization, with B, Fe, and Zn being remobilized mostly from the tip of the leaf, while P and K presented high levels of remobilization mostly from the leaf base. Interestingly, B was remobilized only from the tip and the middle of the leaf blade (Fig. 4a).

The nutrients Ca, S, Mg, B, Mn, and Al showed an accumulation pattern in sugarcane senescing leaves (Fig. 4b). Ca, S, Mn, and Al were accumulated in relatively higher proportions in the leaves. Ca and Al had distinct levels of accumulation in the different portions of the leaf, with levels ranging from 50 to 75 % of accumulation from the tip to the leaf base. Mg and B showed moderate (25 % in average) levels of accumulation. It is also interesting to note that Mg also showed high levels of accumulation in the leaf base, approximately two-fold compared to the other portions of the leaf (Fig. 4b).

The percentages of remobilization or accumulation of nutrients in sugarcane leaves followed the order P > K > N > Cu > Fe > Zn > B and Ca = Al > Mn > S > Mg > B, respectively. The *Pearson*’s correlation matrix between SPAD index and macro and micronutrients contents at the base, middle, or tip positions of the leaf is presented in the Additional file 3. A positive correlation between SPAD index, representing total chlorophyll levels, with remobilized nutrients (N, P, K, Cu, and Zn) was observed, in contrast with a negative correlation with the accumulated (non-remobilized) nutrients (Ca, S, Mg, Mn, and Al). These data represent the pattern of leaf yellowing (Fig. 1B), reduction of the content of the photosynthetic pigments (Chl-*a*/*b* and Cars) (Fig. 1C, D), and the remobilization/accumulation of macro and micronutrients (Figs. 2, 3) triggered by the natural senescence process in sugarcane cv. RB867515.

### Neutral monosaccharides profile

**Fig. 5:**
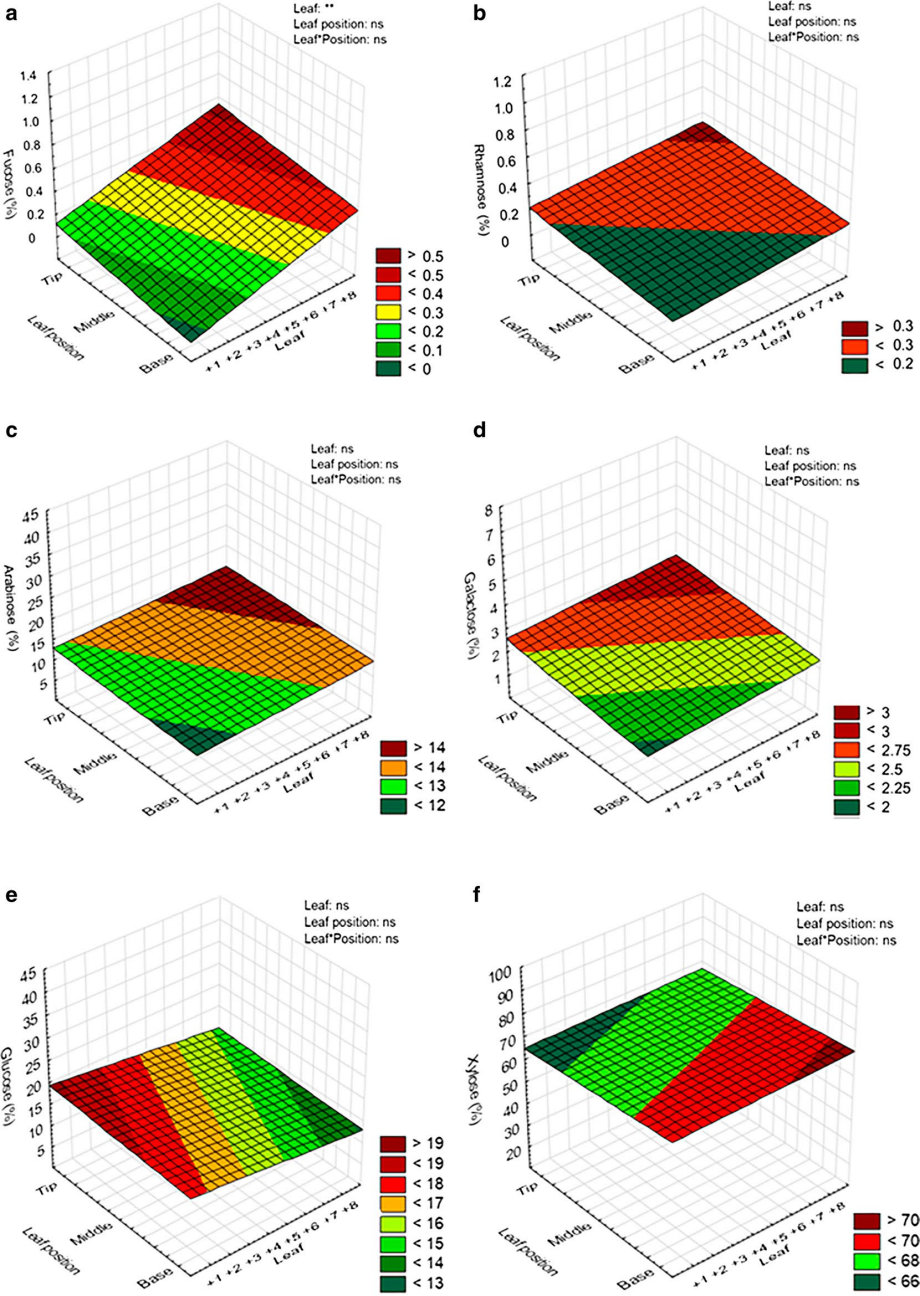
Profile of the neutral monosaccharides fucose (**a**), rhamnose (**b**), arabinose (**c**), galactose (**d**), glucose (**e**), and xylose (**f**) in the ‘between-leaves’ gradient and in the ‘in-leaf’ senescence gradient of the sugarcane cv. RB867515. The “leaf” axis shows the sugarcane leaf number according to Kuijper [26]. The amounts of all neutral monosaccharides correspond to 100 % of the cell-wall major components. Statistical analysis: leaf refers to statistics applied in different leaves (from +1 to +8); leaf position refers to statistics applied to the same leaf (base, middle, and tip portions) and leaf*position refers to statistics applied for the whole data set (‘between-leaves’ and ‘in-leaf’ gradients); asterisk indicates statistical difference: **p ≤ 0.01, *p ≤ 0.05, and *ns* non-significant

The neutral monosaccharides fucose (Fuc), rhamnose (Rha), arabinose (Ara), galactose (Gal), glucose (Glc), and xylose (Xyl) were analyzed from +1 to +8 leaves. Three-dimensional plots were used to represent the levels of these monosaccharides ‘between-leaves’ and ‘in-leaf’ senescence gradients (Fig. 5). Ara and Xyl are monosaccharides released from arabinoxylan, one of the most abundant hemicelluloses in sugarcane cell walls [33]. Both monosaccharides showed no significant differences ‘between-leaves’ and ‘in-leaf’ senescence gradients (Fig. 5c, f), suggesting that arabinoxylan is not degraded in the senescence process. As we used TFA for hydrolysis, we do not expect Glc to come from cellulose. Thus, it is very likely that the detected Glc in sugarcane leaves (Fig. 5e) is derived from the mixed linkage β-glucan (β-glucan), another hemicellulose found in sugarcane cell walls [33]. However, steps to eliminate starch from the cell-wall preparation were not carried out; therefore, starch may also contribute to Glc. In addition, there is a possibility that a minor portion of the Glc and Xyl would come from xyloglucan that also occurs in sugarcane leaf cell walls may not be discarded [33]. A small percentage (~2.5 %) of Gal, and only traces of Fuc and Rha were detected. In these cases, we assumed that these monosaccharides (in addition to some of the arabinose mentioned above) are derived from pectin polymers. Our results indicate that no significant changes in major hemicellulose or pectin cell-wall polymers occur during sugarcane leaf senescence.

**Fig. 6:**
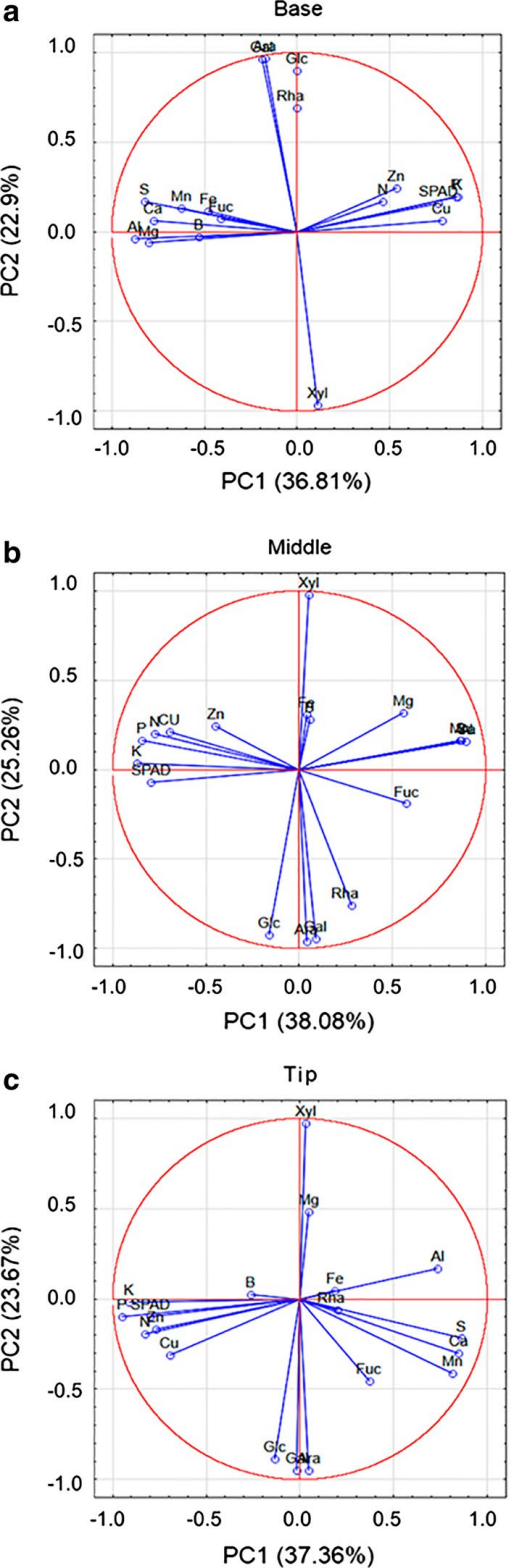
Biplot graphics of the principal component analysis (PCA) from SPAD index, macro and micronutrients, and neutral monosaccharides from base (**a**), middle (**b**), and tip (**c**) portions of the leaf blade. Each point integrates all components evaluated related to sugarcane leaf senescence. Values in *brackets* show the percentage of the variance explained by each axis. *SPAD* SPAD index, *N* nitrogen, *P* phosphorous, *K* potassium, *Ca* calcium, *Mg* magnesium, *S* sulfur, *B* boron, *Cu* copper, *Fe* iron, *Zn* zinc, *Mn* manganese, *Al* aluminum, *Fuc* fucose, *Rha* Rhamnose, *Ara* arabinose, *Gal* galactose, *Glc* glucose, *Xyl* xylose

Co-variance analysis demonstrated that in the ‘in-leaf’ senescence gradient, the monosaccharides Rha, Ara, Gal, and Glc are positively correlated, while Xyl correlates negatively to all monosaccharides listed above (Additional file3). According to the *Pearson*’s correlation matrix between total chlorophyll content (SPAD index), macro and micronutrients levels, and the neutral monosaccharide profile, it was observed that, in general, the sugars correlate only among them, but never with nutrients or senescence (Additional file 3). These results suggest a non-dependent relationship between the composition of the cell wall and total chlorophyll content or nutrient composition during sugarcane leaf senescence. In addition, a principal component analysis (PCA) considering the three parameters mentioned above was performed to analyze the base, middle, and tip portions of the sugarcane leaf blade (Fig. 6). It was noticed that the main differences between senescing and non-senescing leaves are in the total chlorophyll content (SPAD index) and nutrient remobilization/accumulation, and not in monosaccharides levels, supporting the idea that cell wall does not change significantly during sugarcane leaf senescence in cv. RB867515.

### Expression profile of genes involved in cell-wall modification and leaf senescence

**Fig. 7:**
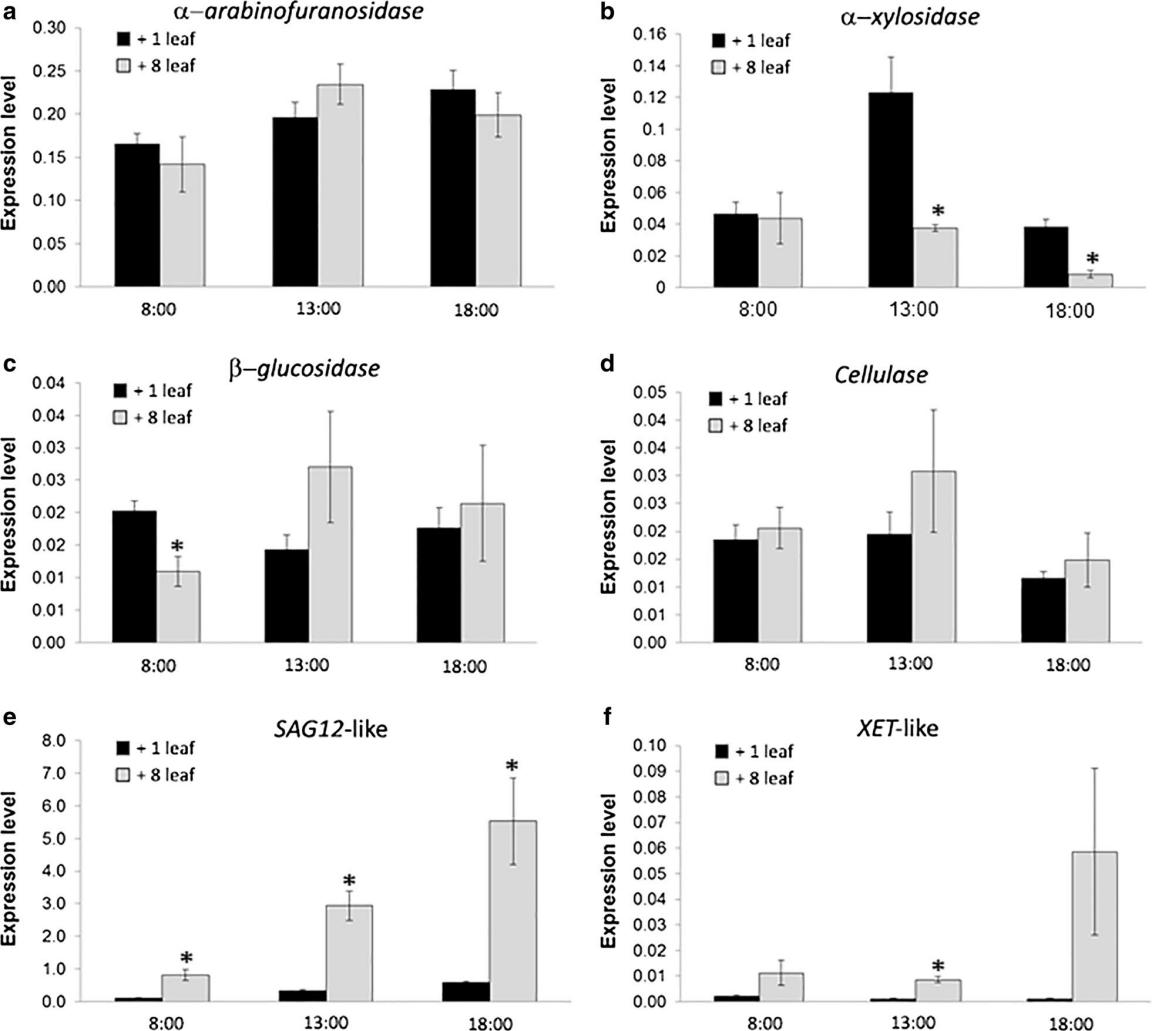
Relative expression of the α-arabinofuranosidase (**a**), α-xylosidase (**b**), β-glucosidase (**c**), cellulase (**d**), *SAG12*-like (**e**), and *XET*-like (**f**) genes in non-senescing (+1, *black bars*) and senescing (+8, *grey bars*) leaves of the sugarcane cv. RB867515. The data were collected at time points from 08:00 to 18:00 h. The *GAPDH* endogenous gene expression was used as reference to measure relative expression levels, which were calculated according to [39]. The *asterisks* indicate statistical significance at *p* ≤ 0.05 when +1 leaves were compared to +8 leaves. *Vertical bars* show ± S.E. for *n* = 3

To evaluate whether subtle changes in cell walls might have occurred during senescence, the expression of the cell wall-associated genes α-arabinofuranosidase, α-xylosidase, β-glucosidase, and cellulase was followed throughout the day (08:00–18:00 h) in +1 and +8 leaves (Fig. 7). The expression of β-glucosidase (Fig. 7c) in senescing leaves was lower when compared to non-senescing leaves in the early morning (08:00 h), while the expression of α-xylosidase was lower in +8 leaves at 13:00 h and at 18:00 h (Fig. 7b). Cellulase (Fig. 7d) and α-arabinofuranosidase (Fig. 7a) expression levels did not significantly change between +1 and +8 leaves during the time period analyzed.

In addition to the cell wall-related genes, we have measured the expression levels of two senescence-related genes: senescence-associated gene 12-like (*SAG12*-like), and xyloglucan endotransglucosylase-like (*XET*-like). The *SAG12*-like gene expression was higher in senescing leaves in all time points analyzed, with levels that were 5.5-fold higher when compared to non-senescing leaves (Fig. 7e). The *XET*-like expression was significantly higher in +8 leaves, when compared to +1 leaves, at 13:00 h (Fig. 7f).

Overall, the expression of some genes related to cell-wall modification suggests a complex regulation of these genes in sugarcane leaves. In addition, senescence-associated genes in sugarcane showed a high level of expression in senescing leaves, and therefore, their promoters are candidates for biotechnological applications aimed at delaying or accelerating senescence in sugarcane.

## Discussion

The first step for 2G-bioethanol production is the hydrolysis of the lignocellulosic material, a task that is not simple to achieve, mainly because of the plant cell-wall recalcitrance. Sugarcane is among the most suitable crops for 2G-bioethanol production. This crop accumulates high contents of sucrose in its culms, which is fermented into bioethanol by yeasts (1G-bioethanol). The remaining biomass from this process composed of sugarcane bagasse, straw, and senescing leaves is considered waste by the sugarcane industry, but may be used for 2G-bioethanol production [1, 4, 42, 43]. The understanding of the natural senescence process and its relationship with cell-wall modification can provide useful information to facilitate 2G-bioethanol generation from sugarcane leaves. In this work, experiments were carried out with a sugarcane commercial variety (RB867515) in field conditions to depict some events occurring during leaf senescence.

### Sugarcane leaf senescence is characterized by strong reduction in photosynthetic pigment content

The sugarcane leaf senescence process corresponds to the last stage in its development, which is governed by developmental aging and influenced by several environmental and endogenous signals, ultimately resulting in programmed cell death [44]. The earliest events during senescence are characterized by chloroplast degradation, loss of Chl-*a*/*b,* and other macromolecules, several changes in cellular metabolism and in gene expression profiles [21, 22, 45]. Chloroplast degradation is usually followed by nutrient remobilization from senescing leaves to other developing organs [27, 28]. Our results demonstrated that photosynthetic pigments content decreased in sugarcane senescing leaves, correlating with the leaf yellowing gradient (Fig. 1).

Chloroplasts are not only essential to photosynthesis, but are an important cellular component for nitrogen (N) storage during leaf expansion [46]. The disassembly of the chloroplasts during leaf senescence releases some nutrients, mainly N, which is mobilized to the sink organs, flowers, and seeds [21]. The optimal utilization of nutrients accumulated during the photosynthetic period is critical for plant fitness, which is critically affected by fine control of leaf senescence process to ensure its effective remobilization [22]. In several higher plant species, leaf senescence progresses from the tip towards to the base portion of the leaf blade [22], indicating nutrient outflow direction [46]. Our results demonstrated that the characteristic senescence pattern observed in other plant species also occurs in sugarcane senescing leaves (Figs. 1, 2, 3).

### Not all nutrients are remobilized during sugarcane leaf senescence

The estimated required nutrients for sugarcane cv. RB867515 (Table 1) predicts that the most required macronutrient for this cultivar is N (14.04 g kg^−1^ dry mass), followed by K (10.87 g kg^−1^ dry mass). Sugarcane N uptake depends on different factors, such as soil, climate, genotype, and biomass accumulation pattern [47]. For instance, sugarcane plants grown in different regions of Australia and Hawaii have higher demand for K than for N [48]. The sugarcane cv. RB867515 is a high-yielding crop, also accumulating high levels of sucrose and biomass, and, therefore, requires a large amount of nitrogen to achieve its highest productivity, suggesting that the requirement for this nutrient is higher in cv. RB867515 than in the Australian and Hawaiian crops mentioned above. Concerning the micronutrients, Fe is the most required (132.67 mg kg^−1^ dry mass), followed by Mn (54 mg kg^−1^ dry mass).

The nutrients remobilized in sugarcane senescing leaves were N, P, K, B, Cu, Fe, and Zn, while Ca, S, Mg, B, Mn, and Al were accumulated (Fig. 4). The levels of N remobilization were higher at the tip and the middle of the leaf blade, the portions where senescence is more pronounced. These results are expected, since most of the N is remobilized from the chloroplast degradation, and this process is also accelerated at the tip and the middle portions of the leaf. The remobilization of P was homogenous throughout the leaf, while K showed higher levels of remobilization in the leaf base. In wheat, it was demonstrated that P and K declined rapidly in the oldest leaf, even prior to senescence [49]. Interestingly, the highly mobile nutrients S and Mg were not remobilized in sugarcane senescing leaves. As discussed by Argenta et al. [27], differently from N, the nutrients S and Mg are stored in the vacuoles under inorganic forms, and their mobilization may be senescence-independent. Our results provide additional argument to this discussion, since the highly mobile nutrients S and Mg showed a small accumulation in sugarcane senescing leaves, instead of remobilized.

Ca, Al, and Mn were not remobilized at high levels in sugarcane senescing leaves (Fig. 4b). These results are expected, because these nutrients are considered immobile in the phloem. Accordingly, Hill and Lonargan [49] showed that Ca and Mn increased during the life cycle of the oldest leaf of wheat and did not decline during senescence. It is known that Al can affect Ca absorption from the soil due to competition or blocking of the channels responsible for Ca transport [50]. Our results showed that the accumulation levels of Ca and Al followed the same pattern throughout the leaf portions, suggesting that Al could be interfering with Ca absorption. However, because Ca contents in the +3 leaves were considered normal (4.6 g kg^−1^, Table 1), one can speculate that, in fact, Al did not affect the absorption of Ca under the conditions used in our experiment, but it can be absorbed from the soil at similar rates of Ca in the sugarcane cultivar studied.

Cu, Fe, Zn, and B were all remobilized in sugarcane senescing leaves. Our data suggest that B might be remobilized only from the tip and middle portions of the leaf, while it was not remobilized in the base. The remobilization of these micronutrients under leaf senescence should be interpreted with caution, since the remobilization could be strongly affected by nutrient deficiency [27]. However, the contents of these nutrients in our experimental conditions were above the critical levels for this crop [47, 51, 52], as demonstrated by cv. RB867515 nutrient leaf index (Table 1). Therefore, it can be suggested that Cu, Fe, Zn, and B are being remobilized in a senescence-dependent manner, and not because of these nutrients deficiency. Our results highlight the intense dynamics of nutrient remobilization in senescing leaves of sugarcane, and these data can be further used to demonstrate the correlations between senescence timing, nutrient-use efficiency, and productivity in this crop [23, 53–57].

### Cell walls are not degraded during sugarcane leaf senescence

Despite the fact that if leaves remained in the field they could return some nutrients back into soil, senescing leaves, given nutrient remobilization, may be better used as feedstock for bioethanol production after suitable pretreatments [58]. One concern about this strategy is the possibility that the plant cell wall could be modified during senescence in ways that deconstruction of senescing plant cell walls would be more difficult. Therefore, here, we aimed at analyzing the neutral monosaccharide profile of sugarcane senescing leaves to evaluate whether substantial changes in-leaf cell wall could occur.

Since no major differences were observed in sugarcane cell walls during the senescence, our results confirm that leaves can be used as feedstock for biofuel production using pretreatments established for non-senescing leaves, without additional efforts. This is extremely important, because currently, at raw sugarcane cutting, 12–20 tons/ha^−1^ of biomass is left on the soil surface, not being used for energy production [59]. About 70 % of this biomass can be used for lignocellulosic ethanol, electricity, and heat production, reducing waste and increasing profits. Moreover, cell-wall composition in leaves of the cv. RB867515 evaluated by monosaccharide analysis (Fig. 5) corroborates the results observed for the cultivar SP80-3280 [33]. This suggests that cell-wall composition does not vary among sugarcane cultivars, facilitating the 2G-bioethanol industrial processing, since little adaptation might be necessary for industrial processing of different varieties.

### Senescence-related genes are strongly expressed during sugarcane leaf senescence and expression levels of cell wall-related genes undergo small changes

Leaf senescence is a well-studied phenomenon in some plant species, especially in *Arabidopsis thaliana*, where molecular and genetic studies have advanced in the past years. Many studies demonstrated that senescence-associated genes (*SAG*s) are involved in Arabidopsis leaf senescence [37, 60–63]. However, a remarkably low number of cell wall-related genes have been reported to be associated to senescence. This corroborates our findings that cell walls undergo very little or no changes during leaf senescence.

Regarding other classes of genes, *SAG12*, specific SAG present in Arabidopsis, encodes a cysteine protease and has high levels of expression during aging and fast response to external factors that accelerate the aging process [64]. The *SAG12* promoter is active when fused to other exogenous genes [61, 65], allowing the target gene transcription specifically during the senescence process. In this work, we were able to find in the sugarcane genome a gene containing a sequence similar to the Arabidopsis *SAG12*, named *SAG12*-like. As expected, our results demonstrated that sugarcane *SAG12*-like was highly expressed in senescing leaves, demonstrating the presence of this senescence-marker gene in the complex sugarcane genome (Fig. 7e).

Park et al. [36] characterized the expression of four genes during leaf senescence in Arabidopsis. One of these genes, *SEN4*, was strongly up-regulated under senescence initiated by several factors and its sequence was similar to *TCH4*, an Arabidopsis gene encoding a xyloglucan endotransglucosylase (XET), currently designated xyloglucan end-Transglycosylase Hydrolase (XTH). XTHs belong to a class of enzymes responsible for complex modifications in xyloglucan molecules being related to its hydrolysis as well as to xyloglucan structure rearrangement through transglycosylation [66]. There seems to exist multiple genes that encode XTHs in sugarcane [67], but their specific functions are unknown. We have chosen to work with *XTH* not only because of its possible involvement in the senescence process, but also because it is related with cell-wall modification, one of the objectives of this study. In fact, the sugarcane *XTH* was up-regulated in +8 leaves (senescing), compared with +1 leaves (non-senescent) in a time-dependent manner. In barley, an endotransglucosylase/hydrolase gene was also found to be highly expressed in senescing leaves of field grown plants [68], reinforcing that *XTH*-like genes could be involved during the senescence process in monocots. Thus, the high differential expression of *XTH* in sugarcane leaves during senescence might mean that xyloglucan (a minor component of sugarcane walls according to Eklöf et al. [66]) could be attacked during senescence. However, here, we did not look into xyloglucan fine structure to determine whether such fine structural changes really occur.

Previous studies have been shown a variety of genes involved in the senescence process in other monocots, such as wheat [69, 70] and barley [68, 71–73]. Kajimura et al. [69] identified two SAG genes in wheat, namely *TaSAG5* and *TaSAG6*, which increased in a time-course manner in the flag leaf and seeds, and, therefore, suggested as molecular markers to evaluate the degree of wheat flag senescence and seed maturation. Gregersen and Holm [70] performed a very comprehensive analysis of the gene expression networks involved in the senescence process in wheat. They found that many genes responsible for the senescence process in wheat overlapped with genes considered to be involved in senescence in other species, but considerable differences were also observed. In addition, it was verified that in wheat, a number of regulatory genes were up-regulated under senescence, especially members of the NAC-domain and WRKY transcription factors. In barley, Parrot et al. [71] applied a steam-girdling protocol in leaves to uncover the gene expression of this monocot plant under senescence. The study demonstrated that many genes for proteases (aminopeptidases, plastidial aspartyl peptidase cnd41, vacuolar thiol and serine proteases, and carboxypeptidase cp-mIII) were differentially expressed, in addition to hexokinases and *SAG*-*12*. A remarkable gene family that is co-regulated with senescence-associated genes belongs to the NAC transcription factor gene family. Christiansen and Gregersen [68] performed a screening of available promoter sequences of barley genes containing DNA-binding motifs for NAC transcription factors, and they found that genes up-regulated during senescence demonstrated a significant representation of these motifs, suggesting regulation of senescence-associated genes by the NAC transcription factors. In addition, this study showed that genes containing the motifs in their promoters were usually highly co-expressed with members of the NAC gene family. Members of the NAC transcription factor gene family were also found in senescing leaves during a transcriptomic study in barley grown in the field supplemented with standard or high nitrogen supply. A detailed characterization of the gene networks involved in the sugarcane senescence process is far beyond of the present work purposes. However, based on the studies mentioned above, a comprehensive gene expression analysis during the leaf senescence process are currently being conducted by our group not only in sugarcane, but also in *Setaria viridis*, an emerging model for genetic studies in C4 plants.

In addition to the neutral monosaccharide analysis, we also characterized expression levels of selected cell wall-related genes. Based on the sugarcane cell-wall composition, we chose four cell wall-related genes encoding an α-arabinofuranosidase, an α-xylosidase, a β-glucosidase, and a cellulase. The expression of these genes was monitored throughout the day (08:00–18:00 h) in non-senescing (+1) and senescing (+8) leaves. Diurnal oscillations in the expression levels are shown in Fig. 7a–d.

Circadian responses of cell wall-related genes are described in the literature [74, 75], although more detailed studies on how these oscillations affect cell-wall dynamics are needed. The α-arabinofuranosidase and cellulase genes did not significantly change their expression levels when +1 leaves were compared to +8 leaves throughout the day, whereas senescing leaves had decreased levels of α-xylosidase and β-glucosidase expression at specific time points. These small changes in cell wall-related gene expression levels reinforce the assumption that the cell wall is not drastically modified under senescence. However, due to the differences observed for α-xylosidase and β-glucosidase expressions between +1 and +8 leaves at specific time points, it cannot be excluded that subtle changes in the cell wall is occurring during the senescence process, but not detected in our analyses due to technical limitations. Therefore, further investigations using more sensitive sugar analysis techniques are needed to shed light on these issues.

## Conclusions

Natural leaf senescence of the sugarcane cv. RB867515 is characterized by significant reduction in photosynthetic pigments content, nutrient remobilization of N, P, K, B, Cu, Fe, and Zn, but not of Ca, S, Mg, B, Mn and Al. Our study on nutrient remobilization under senescence in a vigorous sugarcane cultivar can contribute to the understanding on how nutrient balance in a high-yielding crop is achieved. In general, neutral monosaccharide profile did not change significantly with leaf senescence, suggesting that sugarcane senescing leaves can be used as feedstock for biofuel production using pretreatments established for non-senescing leaves without additional efforts. In addition, the *XET*-like or *SAG12*-like promoters may be used to drive the expression of other stress-related genes or genes involved in cell-wall deconstruction at the onset of the senescence process. Specifically, these promoters could be used to drive the expression of genes related to cell wall-modifying enzymes to produce cell-wall modifications for the harvesting period, facilitating the pretreatments for biomass hydrolysis and the production of 2G-bioethanol.

## Additional files

10.1186/s13068-016-0568-0 Primers used in this study.
10.1186/s13068-016-0568-0 Analysis of variance (ANOVA) of the (**A**) SPAD index, (**B**) macronutrients, (**C**) micronutrients, and (**D**) neutral monosaccharides. The significant data were highlighted in red.
10.1186/s13068-016-0568-0 Correlation matrix between macronutrients, micronutrients, SPAD index, and neutral monosaccharides into in-leaf senescence gradient. (**A**) base, (**B**) middle, and (**C**) tip portions of the leaf blade. Significant correlation data (*Pearson*’s correlation matrix) were highlighted in red (*p* value ≤0.05).

## Abbreviations

2G: second generation
SAG: senescence-associated gene
XET: xyloglucan endotransglucosylase
PCD: programmed cell death
Cars: carotenoids
TFA: trifluoroacetic acid
PCA: principal component analysis
GLM: general linear model
*α*-*ARF*: α-arabinofuranosidase
*α*-*XYL*: α-xylosidase
*β*-*GLU*: β-glucosidase
GAPDH: glyceraldehyde-6-phosphate dehydrogenase
Fuc: fucose
Rha: rhamnose
Ara: arabinose
Gal: galactose
Glc: glucose
Xyl: xylose
