# Supplementary material for: Characterization of sugarcane (Saccharum spp.) leaf senescence: implications for biofuel production
Source: Biotechnol Biofuels. 2016 Jul 22;9:153. doi: 10.1186/s13068-016-0568-0 (PMC4957918; doi:10.1186/s13068-016-0568-0)
Supplement: Supplementary file 1 — 10.1186/s13068-016-0568-0 Primers used in this study. [file 13068_2016_568_MOESM1_ESM.docx]

**Table S1.** Primers used in this study.

| **Oligo** | **Gene name** | **Sequence ID^a^** | **Orientation** | **Sequence (5´-3´)** | **Efficiency (%)** | **Concent. (µM)** | **Reference** |
| --- | --- | --- | --- | --- | --- | --- | --- |
| GAPDH-F | glyceraldehyde-6-phosphate dehydrogenase | SCBFFL4116A05 | Sense | TAACCGAGGTTCACTTGAA | 96.8 | 0.2 | [[42](#_ENREF_41), [43](#_ENREF_42)] |
| GAPDH-R |  |  | Antisense | CCTGTCCTCGTTGACACCAA |  |  |  |
| αAra-F | α-arabinofuranosidase | SCJLLR2013C02 | Sense | ACTGATGATGGGCTTGGATATTATG | 95.8 | 0.6 | [[15](#_ENREF_15)] |
| αAra-R |  |  | Antisense | TTCCATTGTTGAATACCCAGATTG |  |  |  |
| αXyl-F | α-xylosidase | SCJFRZ2029F03 | Sense | CGACGTCCAGCCCTTACTTC | 79.6 | 0.4 | [[15](#_ENREF_15)] |
| αXyl-R |  |  | Antisense | CCACCAACCTCCACCATGAC |  |  |  |
| βGlu-F | β-glucosidase | SCVPRZ2042G06 | Sense | GCCATTGTTCAGCGATGAAGA | 91.8 | 0.4 | [[15](#_ENREF_15)] |
| βGlu-R |  |  | Antisense | TGTCCCAGGGTCCTTCATGT |  |  |  |
| CelS-F | cellulase | SCEQHR1079F11 | Sense | GGGCAGCAAGTTGGCTCTAT | 88.5 | 0.6 | [[15](#_ENREF_15)] |
| CelS-R |  |  | Antisense | GTTGGCCTTCCCCAATCAG |  |  |  |
| SAG12-F | SAG12-like (cistein protease) | AT5G45890 | Sense | AGCAGGAGCTTGTTGACTGTGA | 98.7 | 0.4 | [[38](#_ENREF_38)] |
| SAG12-R |  |  | Antisense | TCGATTCCACCATTGTTGATGA |  |  |  |
| XET-F | XET-like (xyloglucan endotransglucosilase) | SCCCST1002D08 | Sense | ACGACGAGATCGACTTCGAGTT | 97.6 | 0.3 | [[39](#_ENREF_37)] |
| XET-R |  |  | Antisense | TTCCCCTGGCTGTACACGTT |  |  |  |

^a^ Sequences derived from SUCEST (<http://sucest-fun.org/index.php/projects/sucest>; [38]), SAG-12 sequence ID from TAIR.
